# Experiences of cancer care during COVID-19: Phase 1 results of a longitudinal qualitative study

**DOI:** 10.1016/j.ijnsa.2021.100030

**Published:** 2021-05-27

**Authors:** Amanda Drury, Manuela Eicher, Maura Dowling

**Affiliations:** a School of Nursing, Midwifery and Health Systems, University College Dublin, Belfield, Dublin 4, Ireland; b Department of Oncology, Lausanne University Hospital (CHUV), Lausanne, Switzerland; c Institute of Higher Education and Research in Healthcare (IUFRS), Faculty of Biology and Medicine, University of Lausanne, Lausanne, Switzerland; d School of Nursing and Midwifery, National University of Ireland, Galway, Ireland

**Keywords:** Cancer, Cancer care, COVID-19, Distress, Ehealth, Experiences of care, Models of care, Resilience, Telemedicine, Unmet needs

## Abstract

**Background:**

Healthcare services have responded to the challenges of service delivery during COVID-19 with telehealth and hybrid models of care. However, there is limited understanding of the experiences of care amongst people affected by cancer and how their experiences may change and evolve against the shifting landscape of COVID-19 incidence, mortality, vaccination and refinements in service delivery.

**Objectives:**

This study explores the experiences of cancer care amongst people affected by cancer in Ireland during the COVID-19 pandemic. This paper presents the results of the initial cross-sectional semi-structured interviews and the longitudinal qualitative research design which will be employed in this study.

**Design:**

A longitudinal descriptive qualitative study.

**Setting:**

Ireland

**Participants:**

People living with and after cancer or caring for someone with cancer during the COVID-19 pandemic.

**Methods:**

Participants were recruited to the study via social media advertisements and consented to participate in up to three semi-structured interviews between January and July 2021. Initial semi-structured interviews were conducted with 16 participants in January 2021. Participants completed measures of resilience (*2-item Connor‐Davidson Resilience Scale*) and distress (*The National Comprehensive Cancer Network Distress Thermometer)*. Interviews were recorded, transcribed and analysed thematically.

**Results:**

Participants reported low levels of distress and moderate to high levels of resilience on average. Three themes were generated from analysis of the first phase of cross-sectional interviews. Participants described a counterbalance of being cautious of infection and keeping safe through prevention and shielding strategies. Although hospitals felt safe and were working efficiently, some participants felt COVID-19 had compromised person-centredness and empathy in care. While participants valued the measures taken to minimize infection risk, substitution of face-to-face appointments with telehealth services and attending essential face-to-face appointments alone restricted participants' access to professional and social support. Despite this, many participants felt public health measures to reduce transmission of COVID-19 had created a sense of not missing out, feeling safe and reduced difficult social interactions requiring explanation of their diagnosis.

**Conclusions:**

There is an opportunity to learn from the experiences of healthcare delivery from the perspectives of people affected by cancer during the COVID-19 pandemic. The results highlight the complexities and dualities of living with, after or caring for someone with cancer during the COVID-19 pandemic. Opportunities for longitudinal qualitative research to explore the evolving experiences, concerns and persistent and emerging unmet information and clinical needs within the rapidly changing socio-political, socio-cultural and healthcare contexts of the COVID-19 pandemic are highlighted.

**What is Already Known About this Topic**

• The reallocation of healthcare resources in response to COVID-19 have significantly impacted the delivery of healthcare services for chronic conditions***.***
• Delays in diagnostic, treatment and survivorship care pathways contribute to additional distress amongst people affected by cancer.
• There is limited understanding of how experiences of care for chronic conditions may change and evolve against the shifting landscape of COVID-19.

**What this Paper Adds**

• The findings highlight the duality of experiences within and between people living with cancer, a counterbalance of positive and negative impacts of the COVID-19 pandemic on fear and safety, access to support and provision of care.
• While telephone and telemedicine appointments have been implemented to ensure public safety and protection, such models may not adequately address the complex supportive care needs of people living with and beyond cancer.
• This study highlights opportunities for the use of longitudinal qualitative research to explore the evolving experiences of cancer care, within the evolving context of COVID-19.

## 1 Introduction

The COVID-19 pandemic has posed severe challenges to healthcare systems since it was declared an international public health emergency by the World Health Organization in January 2020. To reduce the spread of COVID-19, protect those vulnerable to the risks of COVID-19, and ensure adequate allocation of human resources to manage COVID-19, non-urgent cancer appointments were partially postponed and transitioned to telephone/telemedicine appointments where possible in the initial months of the pandemic (Nekhlyudov et al., 2020; Weinkove et al., 2020, Willan et al., 2020a, Willan et al., 2020b).

People with cancer are considered particularly vulnerable to severe COVID-19 complications (Liang et al., 2020; Yu et al., 2020). The reallocation of healthcare resources in response to COVID-19 have impacted health service delivery for chronic illnesses, including oncology and haematology services (Willan et al., 2020a). One study of haematology activity in a tertiary hospital in the UK between January and April 2020 highlighted reductions in 1) the number of patients referred for specialist haematology review (−57%); 2) the number of patients diagnosed with haematological malignancies (−54%); and 3) patients with haematological malignancies treated with chemotherapy (−24%) (Willan et al., 2020a).

Early reports of cancer patients' experiences of COVID-19 have highlighted a higher perceived risk of infection, fear of infection and more severe complications compared to healthy controls (Casanova et al., 2020). Delays in diagnostic, treatment and survivorship care pathways invariably create additional distress for people affected by cancer. A prospective cross-sectional study from an Italian cancer centre in April 2020 suggested that more than one-third of lymphoma patients self-report anxiety (36%), depression (31%) and post-traumatic stress disorder (36%) (Romito et al., 2020).

One year on from the declaration of the COVID-19 pandemic, there is emerging evidence suggesting variable trajectories of physical and mental health, resilience and life satisfaction outcomes within the general population (Losada-Baltar et al., 2021; Riehm et al., 2021a, Riehm et al., 2021b; Sutin et al., 2021). Studies of the experiences and needs of people affected by cancer during the COVID-19 pandemic have been predominantly cross-sectional (Casanova et al., 2020; Chaix et al., 2020; Romito et al., 2020; Wang et al., 2020). However, cross-sectional studies do not provide an adequate understanding of the impact of rapid changes in national policies to suppress transmission of COVID-19. Longitudinal approaches provide a unique opportunity to understand the perceptions of people affected by cancer with regard to perceived threats of COVID-19 during peaks and troughs in national incidence rates, the rollout of vaccinations, and changes in care arising from transitioning models of healthcare delivery to sustain delivery of healthcare services for chronic illnesses.

Longitudinal qualitative research permits exploration of experiences of change and stability over time (Calman et al., 2013; Saldana, 2003). A longitudinal qualitative approach offers an unprecedented opportunity to understand trajectories of distress, resilience and experiences of care amongst people affected by cancer over a six-month period during the COVID-19 pandemic. A longitudinal qualitative approach will provide insights into the socio-demographic, health systems, public health, political and socio-cultural factors, which may influence levels and trajectories of resilience, distress and unmet needs amongst people affected by cancer during COVID-19. Furthermore, the longitudinal approach may provide additional insight into the experiences of transitions in cancer care during the COVID-19 pandemic, including the transition from treatment to follow-up care. Learning from participants' experiences of adaptations to the delivery of cancer services and the consequences of changes in cancer care during COVID-19 may further support the development and implementation of sustainable and acceptable models of care for people affected by cancer at all points in the cancer continuum.

This longitudinal qualitative study includes three phases and aims to describe the evolution of experiences of cancer care in Ireland during COVID-19 from the perspectives of people living with and beyond cancer. This study will be conducted over a six-month period in Ireland, which will capture information against the changing political and socio-cultural context of COVID-19 in Ireland, including periods of high and low COVID-19 incidence and mortality, changing public health measures to control the transmission of COVID-19, and the rollout of the COVID-19 vaccination programme. This study will:

1 Explore cancer patients' and survivors' experiences of cancer care over a 6-month study period during the COVID-19 pandemic.
2 Understand cancer patients' and survivors' satisfaction with cancer care over a 6-month period during the COVID-19 pandemic.
3 Explore issues influencing cancer patients' and survivors' levels and trajectories of distress and resilience over a 6-month period during the COVID-19 pandemic.

This paper presents the cross-sectional results of the first phase of interviews. This paper presents the longitudinal qualitative research design adopted for this study and will discuss the directions for data collection and data analysis in subsequent phases of the longitudinal analysis. Finally, we discuss the opportunities of a pragmatic longitudinal approach to data collection and analysis of subsequent phases, designed to *build upon* and *enhance* knowledge and understanding of cancer patients' and survivors' experiences of cancer care in Ireland during the COVID-19 pandemic, with a particular focus on the impact of changes in the socio-political and socio-cultural landscape of COVID-19.

## 2 Research methods

### 2.1 Design and participants

This longitudinal descriptive qualitative study invited people living with and beyond melanoma, breast, prostate, lung and colon cancer and their caregivers to participate in up to three semi-structured interviews to understand and describe their care experiences during COVID-19 (Saldana, 2003; Sandelowski, 2000; Sandelowski, 2010). The target population represents the five most common incident cancers in Ireland (National Cancer Registry Ireland, 2020). All participants are adults, aged 18 years or older, receiving systemic anti-cancer treatment or undergoing active surveillance during the COVID-19 pandemic, and able to provide informed consent to participate in telephone interviews.

Recruitment to the initial phase of this study was completed in January 2021. Participants were invited to participate via advertisement on social media platforms. Potential participants were invited to submit an expression of interest to participate in the study. Twenty-two people expressed interest in participating and were emailed a letter of invitation, participant information leaflet and consent form. Two of those who expressed interest did not meet the study inclusion criteria, three did not respond further, and one withdrew before their scheduled interview. Participants who returned a signed consent form were subsequently contacted by phone to arrange a telephone interview at a mutually convenient time. This study received ethical approval from the National University of Ireland Galway Research Ethics Committee (Ref: R20.Jun.04).

### 2.2 Data collection

Sixteen participants consented to participate in up to three semi-structured telephone interviews at T1, T2 and T3, and participated in their first interview between January and February 2021. Interviews were guided by a semi-structured interview schedule exploring participants' perceptions of changes in the continuity of care, perceived risks/benefits of new methods of care delivery, information needs, and worries about cancer relating to COVID-19 (Table 1). An analysis of online narratives regarding COVID-19 in cancer forums provided a framework for this study and informed the development of the T1 interview schedule (Colomer-Lahiguera et al., 2021). The items addressed in the T1 interview schedule will form the basis for interviews at each timepoint to ensure consistency in the study. However, interview schedules utilized at T2 and T3 will include additional items, which will:

1) explore participants' perspectives on the influence of contextual and environmental factors on their distress, resilience and experiences of cancer,
2) interrogate results of previous phases in greater depth,
3) evaluate the evolution of individual concerns and needs over the course of the study period.

**Table 1:** Data collection context, methods and approaches to analysis.

Phase	Time 1 (T1)	Time 2 (T2)	Time 3 (T3)
COVID-19 Socio-political & Socio-cultural context	*January 18th – February 17th, 2021 (Completed):* • High COVID-19 incidence and mortality rates. • National lockdown.	*April 2021 (Projected):* • Reducing COVID-19 incidence and mortality. • Easing of COVID-19 lockdown restrictions. • Vaccination programme underway. • People with cancer receiving initial vaccination.	*July 2021 (Projected):* • Low COVID-19 incidence and mortality. • Restrictions on movement eased. • People with cancer will have completed vaccinations.
Data Collection	*Questionnaire Items:* • Demographic items • The National Comprehensive Cancer Network Distress Thermometer (NCCN-DT) • The 2-item Connor‐Davidson Resilience Scale (CD‐RISC2) *Interview Schedule* • Can you tell me how you feel about your risk of infection? • Can you tell me about your experiences of hospital visits? • Could you tell me about your experiences of support from family/ friends? How has COVID-19 affected that support? • Could you tell me about any support you have received from local cancer support services since COVID-19 began? • Could you tell me about your follow-up appointments? • Could you tell me where you are getting your information updates on COVID-19? • Is there anything that you perceive as a positive change during or since the COVID-19 pandemic? (prompts: regarding your cancer? In general?)	*Pre-Interview:* • Each participant receives a summary of the results of Time 1 thematic analysis. *Questionnaire Items:* • NCCN-DT • CD‐RISC2 *Interview Schedule* • Adapted Time 1 interview schedule; focused on participants perceptions of changes since Time 1 interview. • Feelings about the current national COVID-19 context and its impact on personal well-being and cancer care at the time of this interview. • Personalized questions exploring personal needs and perceived changes in the trajectory of distress and resilience compared to Time 1.	*Pre-Interview:* • Each participant receives a summary of the results of Time 2 thematic analysis. *Questionnaire Items:* • NCCN-DT • CD‐RISC2 *Interview Schedule* • Adapted Time 1 interview schedule; focused on participants perceptions of changes since Time 2 interview. • Feelings about the current national COVID-19 context and its impact on personal well-being and cancer care at the time of this interview. • Personalized questions exploring personal needs and perceived changes in the trajectory of distress and resilience compared to Time 2.
Data Analysis	*Thematic Analysis* Inductive 1) Cross-sectional Thematic Analysis	*Thematic Analysis* Abductive 1) Cross-sectional Thematic Analysis: a Framework b Emergent codes 2) Longitudinal Trajectory Analysis of individual and sub-group data to explore differences in experiences over time, based on trajectories of: a distress, b resilience and c the public health context of COVID-19 from Time 1 to Time 2.	*Thematic Analysis* Abductive 1) Cross-sectional Thematic Analysis: a Framework b Emergent codes 2) Longitudinal Trajectory Analysis of individual and sub-group data to explore differences in experiences over time, based on trajectories of: a distress, b resilience and c the public health context of COVID-19 from Time 1 to Time 3.

During each interview, participants are invited to respond to a brief questionnaire to evaluate their distress, resilience and demographic characteristics. *The National Comprehensive Cancer Network Distress Thermometer (NCCN-DT)* (Roth et al., 1998) is a validated self-report screening tool to measure psychosocial distress in cancer settings (Donovan et al., 2014). The NCCN-DT consists of a one item visual analogue scale (0, "no distress" to 10, "extreme distress") which asks participants to "*Please circle the number [0–10] that best describes how much distress you have been experiencing in the past week including today*."

*The 2-item Connor‐Davidson Resilience Scale (CD‐RISC2)* (Vaishnavi et al., 2007) evaluates participants' resilience via two items: 1) "able to adapt to change" and 2) "tend to bounce back after illness or hardship") and has been validated. Both surveys items have 5‐point Likert scale response (0, "not at all true" to 4, "true nearly all of the time"). An overall CD-RISC2 score is calculated from the sum of both items, which has a possible range of 0–8; higher scores reflect higher levels of resilience.

All interviews at T1 were conducted by a single researcher, an oncology nurse experienced in qualitative research methods and cancer care (MD). This approach serves to enhance relationships and build trust between participants and the researcher, promoting participant retention and enhanced credibility of study findings (Lincoln and Guba, 1985). Fifteen interviews were undertaken by telephone and one by Microsoft teams. Interview times ranged from 12 to 48 min. Interviews were recorded and transcribed verbatim to assist with data analysis. Field notes were recorded immediately after each interview to support data analysis and researcher reflexivity.

Member checking is operationalized within this study through participants' validation of transcripts and reviewing the summarised results of the thematic analysis at each timepoint (Calman et al., 2013). After T1 interviews, all participants received a copy of their interview transcript and were provided with an opportunity to amend or clarify their responses prior to data analysis. At T1, one participant requested that her fears related to COVID be emphasized in the analysis. One participant requested minor changes to her transcript to ensure that she or her family members could not be identified. To enhance the depth of analysis and validation of the study findings, participants will receive a summary of the thematic analysis of interviews conducted at the previous timepoint. Methodological rigour in this study was ensured using strategies to address the credibility, dependability, transferability and confirmability of the research (Table 2) (Lincoln and Guba, 1985).

**Table 2:** Application of qualitative rigour (Lincoln and Guba, 1985).

Criterion	Application
*Credibility*	Research team members experienced in and trained in qualitative research. The use of reflexive journaling and debriefing with the research team promote critical evaluation and reflection on data collection and analysis. Prolonged engagement between the researcher and research participants, building trust and enhancing engagement with interviewees. Recording and verbatim transcription of interviews.
*Dependability*	Transparent reporting of study protocol enables assessment of the research process and future replications of the study. Member checking of interview transcripts and cross-sectional results at each timepoint to support validation of transcripts and study results, promoting more in-depth discussion of cross-sectional findings at subsequent timepoints. Systematic audit trail maintained in NVivo using coding, memo and annotation functions to detail decisions relating to analysis.
*Transferability*	Ecological validation is facilitated at each timepoint, with a description of the social, political and epidemiological contexts of COVID-19 in the Irish context at each of the interview timepoints. Use of participant quotations to illustrate and support analysis and interpretation of the data. Relating findings of the study to existing literature and meta-analysis of results from concurrent cross-sectional studies conducted in Europe and North America.
*Confirmability*	Use of reflexive journaling to explore the influence of the researcher at each step of the research process. Rigorous review of the interview transcripts and analysis according to the Braun and Clarke (2006) Thematic Analysis Framework. Systematic audit trail maintained in NVivo using coding, memo and annotation functions to detail decisions relating to analysis.

### 2.3 Data analysis

Analysis of qualitative data generated within this study is guided by the Braun and Clarke Thematic Analysis Framework (Braun and Clarke, 2006) and managed in NVIVO 12. Aligning with the recommendations of Calman et al. (2013), qualitative data are analysed cross-sectionally at each timepoint to generate a summary of the cross-sectional thematic analysis, which will form the basis for member checking, and inform the interview guide at T2 and T3.

Within this study, time and change are contextual, and against the shifting landscape of COVID-19, are subject to changes in response to shifting political, socio-cultural and healthcare priorities (Saldana, 2003). Consequently, context is a core component of longitudinal data analysis within this study. Longitudinal qualitative data will be analysed within- and between-cases at T2 and T3 (Table 1). Longitudinal within-case analysis will capture changes over time with respect to participants' trajectories of distress, resilience, and healthcare needs and experiences.

Participants' responses to NCCN-DT and CD-RISC items at T1, T2 and T3 will be analysed descriptively to identify sub-groups experiencing high or low distress or resilience at each timepoint and sub-groups who report trajectories of increasing, decreasing or static (high or low) distress and resilience between T1 and T3 (longitudinal). NCCN-DT and CD-RISC data will provide context for longitudinal sub-group analysis of qualitative data to explore the issues discussed by participants which may influence trajectories of distress or resilience longitudinally during COVID-19 (trajectory analysis) (Grossoehme and Lipstein, 2016). Trajectory analysis will also be informed by transitions in the landscape of COVID-19 and social and healthcare policies and mandates over the course of the study to elicit factors which may influence participants' experiences of cancer care and levels of distress and resilience over time. To facilitate longitudinal analysis, time-ordered sequential matrices will be generated to facilitate trajectory analysis exploring the evolution of themes over the timeframe of the study and to support analysis of changes within the context of COVID-19 and participants' distress and resilience over time (Grossoehme and Lipstein, 2016).

As this represents an exploratory study of an emerging phenomenon, the qualitative data from T1 interviews were coded inductively, and the results of thematic analysis of the data are presented in this paper. Subsequent interviews will be analysed cross-sectionally at each timepoint, and codes will be reviewed, organized and reorganized into themes and subthemes. The coding framework developed at T1 will provide a framework for cross-sectional analysis at T2 and T3. Where new codes are identified at subsequent timepoints, they will be assigned new codes and assimilated into analysis at each timepoint. In the final stages of analysis, data will be reanalyzed deductively, cognizant of themes identified in the longitudinal analysis, to ensure they represent participants' contributions. Themes that are insufficiently supported by the data will be discarded, and each theme will be defined, named and written up.

## 3 Results

### 3.1 Sample characteristics

Sixteen participants were recruited to this study and interviewed in January 2021 (T1). All consented to be interviewed again at T2 and T3. One participant was male. The majority of participants had a diagnosis of breast cancer (*n* = 12); the remaining participants had a diagnosis of colorectal cancer (*n* = 3), and one person was caring for a family member who had a diagnosis of lung cancer (*n* = 1). The majority of participants were diagnosed between six and twelve months prior to the commencement of T1 interviews (*n* = 10), and nine reported they were in remission from cancer at the time of T1 interviews (Table 3).

**Table 3:** Participant characteristics.

Characteristic	Response	*n*	*%*
*Participant*	A person with cancer	15	94%
	Family member and caregiver	1	6%
*Age groups*	18–40	5	31%
	41–60	10	63%
	61–80	1	6%
*Gender*	Female	15	94%
	Male	1	6%
*Living arrangements*	Living with family member(s)	15	94%
	Living alone	1	6%
*Diagnosis*	Breast cancer	12	75%
	Colorectal cancer	3	19%
	Lung cancer	1	6%
*Time since first diagnosis*	6–12 months	10	63%
	1–2 years	1	6%
	2–3 years	1	6%
	3–4 years	1	6%
	4–5 years	1	6%
*Disease Status*	Receiving treatment	5	31%
	Recieving treatment for recurrent disease	2	13%
	In remission	9	56%

### 3.2 Socio-political and socio-cultural context of COVID-19 in ireland during T1 interviews

T1 interviews commenced on January 18th, 2021. At the time of T1 interviews, Ireland had the highest rate of COVID-19 infections internationally and had entered its third lockdown since March 2020. Acute hospitals across Ireland were in surge capacity, and mortality rates were the highest since the pandemic began. A new, more transmissible strain of COVID-19 (VUI-202,012/01) accounted for approximately 60% of incident cases at the time of T1 interviews. The nation's mood had shifted to fear; anecdotal reports from cancer services highlighted increasing demand for psychosocial cancer support (Greally, 2021). During the T1 data collection period, daily mortality reached the highest point in Ireland since the pandemic began (February 2nd, 2021; *n* = 101), and the government announced further travel restrictions, including curtailment of overseas travel.

Against the backdrop of increasing incidence and mortality, the rollout of the COVID-19 vaccination commenced in late December 2020. By the time of the final T1 interview on February 17th, 2021, people over the age of 85 living in the community were beginning to receive their first vaccination. By this time, the number of people with COVID-19 in intensive care units was also declining. However, Irish news media was beginning to describe concerns about the longer-term impacts of successive lockdowns, including the potential 'cancer tsunami' which would result from delays in people seeking care for potential signs and symptoms of cancer.

#### 3.2.1 Distress and resilience

On average, CD-RISC2 scores indicated moderate to high levels of resilience within this sample ($\bar{\chi }$=6.5; SD=1.6, range=2–8) (Table 4). Fourteen participants stated that they were able to adapt to change and bounce back after illness or hardship, very often or nearly all the time (88%). Distress scores within the sample were low on average ($\bar{\chi }$=3.4; SD=2.2, range=1–9). Four participants reported moderate levels of distress, eight reported low levels of distress, and three participants reported no distress.

**Table 4:** Summary of distress and resilience outcomes.

Outcome	Mean	*SD*	Median	Range
***Resilience***				
*I am able to adapt to change*	3.3	0.86	3	1–4
*I tend to bounce back after illness or hardship*	3.3	0.86	3	1–4
*CD-RISC2*	6.5	1.59	6	2–8
***Distress***				
NCCN-DT	3.4	2.22	3	1–9

#### 3.2.2 Thematic analysis

Three themes were generated from T1 interviews describing the experiences of COVID-19 and cancer care from the perspective of people living with and affected by cancer, 1) Being careful, keeping safe and feeling safe; 2) Shrinking supports and feeling isolated; and 3) Not missing out.

##### 3.2.2.1 Theme 1: being careful, keeping safe and feeling safe

Participants recognized their increased risk of infection and endeavoured to minimize their risks of contracting COVID-19 through shielding and cocooning measures, avoiding public places, even for essential purposes such as shopping. Participants described the measures taken by all members of their household to minimize risks of introducing COVID-19, including minimizing contact with others from outside the household, cleaning packaging and groceries as they enter the home from outside, and family members avoiding work, school or other social activities associated with greater risk of contracting COVID.

*I was very careful I mean I didn't go into a shop from March until … probably October or November even. (4)*

*I was terrified. I was terrified. No other way of describing it, [laughing] our shopping would be delivered, and I hated shopping day because we had to clean every item down before it came in. (5)*

*[…] they [children] wanted to go out and play with the kids out the back, as time went on it was hard to keep them in. We were trying our best to keep them away from other people. We were told you know that was important. But that was the biggest challenge as opposed to anything else. (3)*

*There is a massive risk coming into our house every day but we're very lucky, we're clean, we have to extra sanitize and to do 3 h cleaning extra that I never did before. And sometimes you just, you have no choice. I can't bring anyone in, I have no one to help me, I have no support, my family don't live near me. And I can't risk a friend of mine coming in case she's been around someone. So you're very much on your own all of the time, you suffer in silence because you know it will end. (7)*

Despite implementing shielding measures, for some participants, admission to hospital or attendance at hospital appointments was unavoidable. Several participants described anxiety and distress at the prospect of attending these appointments and being admitted to hospital.

*I probably was a bit more concerned when I was in hospital after the surgery as well, just you know, even within the ward. None of the patients were wearing masks and you know there was a lady in the bed, you know in the next bed to me who had a cough you know, not COVID but just, you know, things like that. I think you know, would make you a bit more on edge, which if it was another year you probably wouldn't think anything of it. But again, you know it's only smaller things like that. (14)*

Those who attended hospital appointments spoke positively of safety measures taken by hospitals to protect patients, including pre-appointment COVID questionnaires, use of personal and protective equipment, and visibility of cleaning and disinfection in public areas. One participant suggested fears about COVID-19 in hospitals was the uncertainty about how compliant other patients were with personal protective equipment and public health guidelines to minimise the spread of COVID-19. However, many suggested that a benefit of the restrictions imposed by COVID-19 was that few patients were attending the hospital, reducing delays during appointments. Some participants alluded to their awareness of others who experienced cancellations and delays in treatment and their concerns about access to systemic treatment and delays for surgery. However, no participant reported experiencing treatment delays or altered treatment regimens as a result of the pandemic.

*I felt safe enough, they [hospital staff] were all wearing their masks and their gloves, and I found the whole place spotless as well. I had no trouble that way, no worries at all […] it's [COVID] had made people more hygienic you know what I mean, more cleaning I suppose compared to maybe when I thought about hospitals before they are more sterilized now. (2)*

*If anything, I'd probably nearly feel more nervous of other patients, or you know, sometimes some people can be a bit more lax you know. I suppose with how they wear their masks. […] I've seen the difference since I initially went to the hospital and went to the various clinics; just you know, it was unrecognizable. I suppose just the amount of people who were in the clinics, you know, at that stage compared to what they were later in the year. (14)*

*I did feel very safe like, all the social distancing, the nurses would ring you like regarding COVID before you went in and like when you got there you were tested and brought in different doors. You felt very, very safe. So then it was just a matter of getting your chemo and getting home. (12)*

*[…] you feel that you are prioritized more and it's a more organized system in place perhaps to get things moving through. (3)*

*He was in hospital twice and each time was for about a week, it really took its toll mentally on him. Because again, the symptoms he had, he had to be isolated. And you know, simple things like being able to work the television, you know and he didn't have, you know just the company or the chitchat or whatever. (15)*

##### 3.2.2.2 Theme 2: shrinking supports and feeling isolated

The experience of being alone for hospital visits at critical points in their diagnosis, treatment and follow-up care was described by several participants. While participants noted efficiencies in cancer services, several described the isolation, loneliness and distress of attending hospital appointments without their support networks.

*I'd nobody with me, so nobody heard [the diagnosis], you know my husband wasn't there so I phoned him when I was outside and I was bawling on the phone, sure he thought I was going to die […] (9)*

*Then with radiotherapy you'd have to, you'd arrive, you'd have to ring and wait outside and be called in have your temperate taken. Go back to the car and wait in the car until you were called to go into radiotherapy. And then you wouldn't meet anyone until you went in, you met your radiation therapist. They were cleaning down when you went in which was comforting, but I had to go on my own. Nobody could travel with me because we were in, it was in lockdown. And so, I found it lonely in the car on my own. I found my first session really, really stressful. I was crying coming home and I was never going back again. I found, I hated it, I hated every minute of my radiotherapy. (5)*

Two participants described at least one healthcare professional that was accessible to them, or provided additional support beyond what they expected, by staying with them for company prior to surgery. However, many narratives highlighted how COVID-19 dominated the cancer care agenda during appointments and when seeking advice, restricting opportunities for engagement with healthcare professionals and the person-centredness of care. Some believed that healthcare professionals' preoccupation with COVID-19 compromised empathy and care, acting as a barrier to care and support during treatment.

*[…] in the last year and a half when I was first diagnosed, I noticed the empathy has changed completely since COVID. There's a lack of empathy. There is very much a lack of communication from the hospital to the patients. And really without sounding very dramatic, there's a lack of care at the moment because they seem to presume that COVID only exists now, even in the hospitals its very much well COVID, well COVID, well COVID […] it's like they don't want to communicate, they don't feel they need to now, because COVID has taken over from their care, so they can use any excuse they want to now and they can blame it on COVID. (7)*

*Trying to ring and just get information about how he was, it was very difficult. And even there was one instance where he rang me, and he was sitting in a chair for hours because there were no beds […] or he'd ring me because he was freezing cold in a place where he was, and I'd have to try and ring to see was there anyone that could give him a blanket. So there was those two bits, I suppose, out of the whole year, that you know it just, it was very frustrating. But again, you know, look it I understand, I know myself the way things work. (15)*

For participants whose appointments had transitioned to telephone consultations, they understood the importance of this measure to limit the number of people visiting hospitals. However, the transition to telephone consultations for some participants was an added barrier to support and limited person-centeredness in care interactions. Participants' narratives suggested that these transitions in care required them to be more persistent to obtain information and supportive care to manage symptoms and concerns during treatment and follow-up care. Most participants used online support groups and resources for information. However, not all found online resources reliable and relied heavily on their oncology team for information.

*I just feel at the moment that I'm not being listened to, you know, one of my phone consultations back during COVID lasted 2.5 min. (1)*

*[…] I suppose it's nicer to sit there with the consultant because I suppose you have more of an opportunity to, to you know I suppose go into things with them you know if you need to. The face-to-face I suppose is a bit more personal always isn't it. So, you know I had missed that a little bit but look, I understand the way things are, and you know it's for our own safety, and they don't want to be bringing people in needlessly like, you know. (13)*

*[…] it's not ideal I guess in a way you kind of need to be maybe a bit more prepared, I think because on the phone it's very easy to just go, oh yeah everything, grand, and then the phone call is over in literally a minute or two and then you are like, oh I forgot to ask them that, or I didn't bring that up or whatever. I think you know when you are face-to-face, it's more of a natural conversation, it's more, you know, you are more inclined to maybe talk about things maybe a little bit more. (4)*

*COVID hasn't reached my hospital yet, but yet any time I enter the hospital or speak to a nurse, they're very much like, they bring in the COVID line, which doesn't, it doesn't wash with me as a patient. They try to insult your intelligence by thinking that this will take back, you know, to times where you'd ring with a sore pain, or you'd ring with the side effects that they want you to ring with. But now, you don't get a call back for two days, even though COVID is not in their hospital. That's what I see a lot of. (7)*

*I actually called them up [the cancer care team] for the first time in a long time about the vaccines because I was a bit concerned about any interaction between Tamoxifen and the vaccines, and you know, between that and the fake news that you hear like I just wanted more advice. And so, I left a voicemail, and within two days, she got back to me; we had a long conversation. So, no, I do feel, you know, I feel quite well supported by them. (10)*

Several participants also acknowledged how the variety of peer support and cancer support services that would be available under normal circumstances were no longer accessible to them. Some of those who had received cancer treatment before the pandemic contrasted the opportunities to speak with other patients and have family members present with them during appointments, with the isolation and loneliness of appointments where they attended alone or were distanced from other patients. While family members were acknowledged as a critical source of support, the shrinking network of support meant that family members assumed a disproportionate burden for support and care of the person with cancer, both due to reduced formal supports and isolation from wider family and social networks.

*[…] when it [radiotherapy] started, I found it very lonely because I was used to in chemo, my mother would come with me to all my chemo sessions. And there would be in [hospital] there's four patients sit around a table, and their family members can sit with them, we used to have a great laugh at chemo. […] (5)*

*[…] you actually don't get the chance to chat to anybody. That's the only, I suppose, negative side of COVID; you don't get anyone to talk to through the whole thing. (8)*

*I suppose the one thing that I found difficult with it all, because of COVID, it was difficult to reach out for support from a mental health point of view, because normally you would have [cancer support centre]. And they shut down kind of petty much completely other than you know phone support. But they offer a whole range of other services, and I wasn't able to avail of any of those and they also have services for children and partners as well. None of that was available. (11)*

*We did as much as we could* ... *I'd leave; you know, medicines, or whatever, at the door, and she would kind of, she would be up at night with him you know when he was sick. (15)*

##### 3.2.2.3 Theme 3: not missing out

Despite the challenges described by participants, many described a sense that they were not missing out due to COVID-19, as everyone had to live within the same restrictions. Many felt that COVID-19 had introduced a new-found perspective on life, whereby they appreciated the opportunity to foster closeness with their families. Despite the isolation associated with cocooning, several participants suggested that staying at home removed social confrontations prompted by their appearance, which placed pressure on them to explain their diagnosis. This gave participants a sense of control over the disclosure of their diagnosis, which otherwise would have been visible to the public.

*It's just being close to them, so the positive I can take is just, kind of, figuring out what's really important. And its family and just being able to kind of spend time with them while I can. It's been cruel. Just the distance and looking in the window when someone is so sick and not being able to, the helplessness not being able to do anything about it for fear. (15)*

*I suppose everybody is in the same boat. I feel I'm not missing out as much. (16)*

*[…] I've been saved because of COVID, from walking down the streets and having 20 million people looking at my baldy head and my no eyebrows; and ask me the question, and have to deal with the 'oh I'm so very sorry to hear that,' etc., etc., etc. Which I can imagine, at the start, when you're told you have cancer, every time that you have to tell someone else, it's like hearing the news all over again. So, I think in that sense, because of COVID, I was saved a lot of that. I was able to do a lot of it on my own terms. I was able to manage the flow of information. (6)*

*Everybody keeps looking at me when, you know, when I was completely bald, and I was thinking, well I don't have to go anywhere so nobody else seen me [laugh]. (9)*

### 3.3 Summary of qualitative findings

The T1 interview findings reveal that irrespective of being on treatment or in follow-up, all participants were fearful of contracting COVID-19 and took measures to stay safe. They valued the restrictions being taken in their treatment centres and appreciated that follow-up had to shift away from clinic visits. However, for some, the lack of face-to-face consultations affected support systems and opportunities to ask questions. Moreover, two participants strongly believed that COVID-19 was being used as an excuse by healthcare professionals to minimize interactions with patients and avoid intimacy and relationship building. Not being allowed to have a family member with them during treatment was a source of isolation for participants having treatment. The loss of wider support services in the community was also isolating for many. However, a sense of 'not missing out' was strongly expressed across the interviews with everyone else also staying at home and restricted in their movements. Finally, not having to face the public and explain their cancer diagnosis brought relief to some.

## 4 Discussion

This study presents a snapshot of the perceptions and experiences of people with cancer about their care and support during a period of high incidence, high mortality and national lockdowns in Ireland in 2021. Participants' narratives of their perceptions of COVID-19 reflect their recognition of increased risks of severe COVID-19 complications amongst people with cancer (Casanova et al., 2020; Liang et al., 2020; Yu et al., 2020). Participants' discussion of their perceived risks and fears related to COVID-19 were punctuated by discussions of strategies to reduce the risk of contracting COVID-19 and reflect the findings of quantitative studies of people affected by cancer conducted during the initial wave of COVID-19 in Europe (Casanova et al., 2020; Chaix et al., 2020; Romito et al., 2020; Wang et al., 2020). Congruence in the results of studies conducted at sequential points during the COVID-19 pandemic may reflect parallels in the socio-political and socio-cultural contexts and widespread public apprehension and uncertainty within successive waves of the pandemic. Nevertheless, despite participants' concerns regarding COVID-19, the majority reported high levels of resilience, while more than two-thirds of participants reported no or low levels of distress.

This study adds to the existing evidence regarding the impact of COVID-19-related curtailments in cancer services (Romito et al., 2020; Willan et al., 2020a). While restrictions in cancer services may contribute to distress amongst people with cancer, it is difficult to determine whether distress is a consequence of perceived risks of COVID-19, shortcomings in access to supportive care services, or a combination of both (Romito et al., 2020; Willan et al., 2020a). This study suggests that the strategies implemented by healthcare organizations to reduce risks of transmission were welcomed by cancer patients and contributed to feelings of safety when attending clinical settings when required. However, many described counter-concerns about restrictions in access to professional and peer support and a perception that COVID-19 was a scapegoat for shortcomings in cancer-related care, particularly related to communication and person-centred care.

Prior to the pandemic, eHealth interventions, including telemedicine, videoconferencing and telephone support were identified as an emerging innovation with the capacity to address anticipated demands for healthcare associated with ageing, multimorbidity and workforce shortages (European Commission, 2012; van Gemert-Pijnen et al., 2012). Indeed, eHealth interventions appear to enhance access to care and support improved outcomes amongst people affected by cancer. However, there is limited evidence regarding the clinical effectiveness of eHealth interventions (Haase et al., 2020). In studies of cancer patients' satisfaction with telemedicine appointments in the first months of the pandemic, on average, patients were satisfied with healthcare (Layfield et al., 2020; Palandri et al., 2021). However, less than one-third of haematology patients indicated they would be willing to continue telemedicine appointments for routine care beyond the pandemic (Palandri et al., 2021). The results of this study add to this evidence, suggesting telemedicine may not provide sufficient access to professional and peer support for people with cancer. However, the onset of the pandemic provides a unique opportunity to learn from the rapid implementation of telemedicine services, which can inform the design and adaptation of models of care in the longer-term which are responsive to the needs of people affected by cancer and which facilitate access to high-quality cancer care beyond the pandemic.

Longitudinal qualitative research approaches present a unique opportunity to explore the evolving experiences, concerns and persistent and emerging unmet information and clinical needs of people affected by cancer during the COVID-19 crisis, including changing attitudes and perceptions of care. A longitudinal approach provides opportunities to obtain insights into long-term sources of distress and unmet need which persist as the pandemic progresses, and potentially beyond the pandemic as healthcare services adopt new approaches to healthcare delivery in the aftermath of COVID-19. Integration of quantitative measures of distress and resilience within this study will permit a more in-depth analysis of issues that may affect participants' trajectories of distress and resilience during the period of this study. In addition, the longitudinal approach adopted in this study will permit contextualized approaches to thematic data analysis, considering the influence of socio-political and socio-cultural factors, including stabilizing incidence and mortality rates; rollout of vaccinations to people vulnerable to complications of COVID-19; and refinements in service delivery models in response to emerging evidence, risk and preferences of people affected by cancer.

### 4.1 Reflections on the limitations of longitudinal qualitative approach

While longitudinal qualitative methods offer an opportunity to understand trajectories of distress, resilience and experiences of care amongst people living with cancer, it is not without limitations. Recruitment, retention and sustained engagement are core considerations in the design of longitudinal qualitative research studies (Calman et al., 2013). While six months may be considered a short timeframe for a longitudinal qualitative study, the timing of data collection was selected to minimize the duration of commitment required from participants and capture anticipated developments in the COVID-19 context in Ireland, including periods of high and low incidence and mortality and the vaccination rollout trajectory. To support retention and engagement of participants over the six-month period of data collection, participants will receive 1) a copy of their transcripts four weeks after each interview, and 2) a summary of the thematic analysis of interviews between seven and ten weeks after their interview. Sharing the thematic analysis with participants at this time will allow each person between two and four weeks to read and consider the results of the previous round of interviews before they participate in their next interview. This strategy supports participants' sustained engagement according to their preferences and the development and validation of thematic findings. Furthermore, the sharing of cross-sectional findings with participants will facilitate a more in-depth exploration and critique of common themes at subsequent interviews, as participants will be given time to review and reflect on findings from previous timepoints. Reflective notes kept at the time of the first interview were also useful to maintain a record of key aspects of discussion within individual interviews which will be explored in subsequent interviews.

A significant limitation of this study is the representation of women (*n* = 15) and people living with and after breast cancer (*n* = 12) within the sample, and the under-representation of caregivers (*n* = 1). As a result, the findings of this study may not be representative of the experiences and needs of people living with or after or caring for people with other forms of cancer. While this study aimed to recruit a heterogeneous sample of people living with and after cancer and caring for people with cancer, the sampling strategy is limited by the online approach to recruitment and constraints of self-selection. Furthermore, while this study will collect quantitative data regarding participants' resilience and distress, this data will be used to characterize the sample and provide context for cross-sectional and longitudinal analysis of qualitative data. Due to the small sample size, the results of descriptive statistical analysis are not generalizable.

## 5 Conclusion

The cross-sectional results of the first phase of this longitudinal qualitative research study provide insight into the complexities of living with or after cancer during the COVID-19 pandemic. This study highlights opportunities for the use of longitudinal qualitative research to explore the evolving experiences, concerns and persistent and emerging unmet information and clinical needs within the rapidly changing socio-political and socio-cultural context of the COVID-19 pandemic. The findings highlight the duality of experiences within and between people living with cancer; a counterbalance of positive and negative impacts of the COVID-19 pandemic; of feeling afraid and feeling safe; of new-found efficiencies in services with shortcomings in access to holistic care and support. Telephone and telemedicine services have been implemented rapidly to ensure the safety and protection of people with cancer against COVID-19; however, findings from the initial phase of this study suggest that such models may not adequately address the complex supportive care needs of people living with and beyond cancer. There is an unprecedented opportunity to learn from the experiences of healthcare delivery from the perspectives of people affected by cancer during the COVID-19 pandemic. This knowledge has the potential to inform the design and adaptation of systems of care in the longer-term which can facilitate access to and quality of care beyond the pandemic.

## Declaration of Competing Interest

AD, ME and MD report no conflict of interest. The authors alone are responsible for the content and writing of this paper.
